# Increasing mortality in the United States from cholangiocarcinoma: an analysis of the National Center for Health Statistics Database

**DOI:** 10.1186/s12876-016-0527-z

**Published:** 2016-09-21

**Authors:** Kaelan J. Yao, Salma Jabbour, Niyati Parekh, Yong Lin, Rebecca A. Moss

**Affiliations:** 1 West Windsor Plainsboro High School South, Plainsboro, NJ 08550 USA; 2 Division of Radiation Oncology, The Rutgers Cancer Institute of New Jersey, New Brunswick, NJ 08901 USA; 3 College of Global Public Health & Population Health, Langone School of Medicine, New York University, New York, NY 10003 USA; 4 Biometrics Division, The Rutgers Cancer Institute of New Jersey, New Brunswick, NJ 08901 USA; 5 Division of Medical Oncology, The Rutgers Cancer Institute of New Jersey, New Brunswick, NJ 08901 USA

**Keywords:** Cholangiocarcinoma, Mortality, Time trends

## Abstract

**Background:**

While mortality in the United States has decreased for most cancers, mortality from combined hepatocellular liver cancer and intrahepatic cholangiocarcinoma (ICC) has increased and ranked 1st in annual percent increase among cancer sites. Because reported statistics combine ICC with other liver cancers, mortality rates of cholangiocarcinoma (CCA) remain unknown. This study is to determine CCA mortality trends and variation based on national data.

**Methods:**

This nation-wide study was based on the underlying cause of death data collected by the National Center for Health Statistics (NCHS) between 1999 and 2014. The Center for Disease Control (CDC) Wide-ranging Online Data for Epidemiologic Research (WONDER) system was used to obtain data. ICC and extra-hepatic CCA (ECC) were defined by ICD-10 diagnosis codes. Age-adjusted mortality rate was standardized to the US population in 2000.

**Results:**

There were more than 7000 CCA deaths each year in the US after 2013. CCA mortality for those aged 25+ increased 36 % between 1999 and 2014, from 2.2 per 100,000 (95 % confidence interval [CI] 2.1–2.3) to 3.0 per 100,000 (95 % CI, 2.9–3.1). Mortality rates were lower among females compared with males (risk ratio [RR] 0.78, 95 % CI 0.77–0.79). Asians had the highest mortality. Between 2004 and 2014, the increase in CCA mortality was highest among African Americans (45 %) followed by Asians (22 %), and whites (20 %).

**Conclusion:**

Based on the most recent national data, CCA mortality rates have increased substantially in the past decade. Among different race/ethnic groups, African Americans have the highest increase in CCA mortality.

**Electronic supplementary material:**

The online version of this article (doi:10.1186/s12876-016-0527-z) contains supplementary material, which is available to authorized users.

## Study highlights

### What is current knowledge

While research has begun to focus on the increasing incidence of intrahepatic cholangiocarcinoma (ICC) as a distinct entity from hepatocellular carcinoma, the overall mortality from cholangiocarcinoma (CCA) in the United States beyond 2005 has not been reported.

### What is new here

Our study of the National Center for Health Statistics demonstrates that the death toll due to CCA rose substantially in the past decade in the US and has exceeded 7000 annually, which is more than double the widely-quoted American Cancer Society estimate of 2000–3000 new cases of CCA per year. Among different race/ethnic groups, African Americans have the highest increase in CCA mortality.

## Background

Cholangiocarcinoma (CCA), also known as bile duct cancer, is a rare cancer originating from the epithelial cells of the biliary ducts [1]. CCA can occur anywhere along this tract from the ampulla of Vater to the intrahepatic biliary radicals. The hepatic duct bifurcation is historically reported to be the most frequently involved site; the extrahepatic cholangiocarcinoma (ECC) tumors at this particular location are called Klatskin tumors [2]. If CCA occurs within the intrahepatic biliary radicals, it is termed intrahepatic cholangiocarcinoma (ICC). Clinical presentation is variable and dependent on the location of the primary tumor, and it is rare for symptoms to manifest early in the course of the disease [3].

Risk factors for CCA include primary sclerosing cholangitis, bile duct stones, liver fluke infection, biliary-duct cysts, hepatolithiasis, inflammatory bowel disease (IBD), hepatitis C, Hepatitis B, cirrhosis, obesity, diabetes, alcohol, smoking, and genetic polymorphisms [4–7], all associated with inflammation [6, 8]. CCA is associated with high mortality. The median overall survival is 20–28 months and 5-year survival rates are about 25 % [9, 10]. Given the rising incidence and high mortality, a better understanding of the populations at risk for mortality from CCA is warranted.

Historically, the Surveillance Epidemiology, and End Result (SEER) classification system placed ICC in the same category as primary liver cancer of hepatocyte origin, hepatocellular cancer (HCC) [4]; however, given the neoplastic origin of ICC in the biliary ducts, it should be studied together with ECC for purposes of understanding risk factors or mortality. Accordingly, the term CCA is now used for all primary tumors of the bile ducts [11]. Because the SEER database combined ICC with HCC [4], and because ECC has historically been reported separately, comprehensive national mortality rates of CCA are not previously well-characterized. While cancer mortality decreased in the US overall by 1.5 % per year during 2008–2012 [12], during the same period the mortality rate from cancers of the liver (HCC and ICC) increased by 3.3 % per year [12]. The relative change was the highest found among the 19 cancer sites tracked [12]. However, these results do not tell us if the steep increase arose from changes in HCC, ICC, or both.

Existing reports documented 300–400 % increases in CCA mortality rates in the US between 1975 and 1997 [13]. A study specifically of ICC published in 2014 found that the incidence of ICC in the US had increased between 1973 and 2010 [14], but did not include ECCs [14]. Although a recent study has examined incidence of both ICC and ECC in the SEER database [15], CCA mortality for the entire US population beyond 2004 has not been studied; therefore it has not been shown if CCA mortality continues to rise. To provide contemporary insight into CCA mortality rates, we undertook a national study based on ICD-10 cause of death, the first classification scheme to provide distinct codes for intra-hepatic CCA (ICC) and extra-hepatic CCA (ECC), thus allowing calculation of overall CCA mortality. Using this data, we sought to examine CCA mortality over time and to assess the effects of age, gender, and race or ethnicity.

## Methods

### Data sources

The data for this study came from the underlying cause of death data collected by the National Center for Health Statistics (NCHS), which comprises data for the entire US population. Data for US residents over age 25 were extracted from the Centers for Disease Control (CDC) Wide-ranging Online Data for Epidemiologic Research (WONDER) system to describe the CCA mortality rates between 1999 and 2014. Data for the population under age 25 were unreliable due to the small number of events and therefore were excluded from the analysis. ICC was defined by ICD-10 code C22.1 (Intrahepatic bile duct neoplasms) and ECC was defined by ICD-10 codes, C24.0 (extra-hepatic bile duct neoplasms), C24.8 (Overlapping lesion of biliary tract neoplasm), and C24.9 (biliary tract, unspecified neoplasms). Informed consent was waived as the raw data are publicly available via CDC WONDER. The codes used to identify ICC and ECC are provided in the method section.

### Statistical analyses

**Fig. 1:**
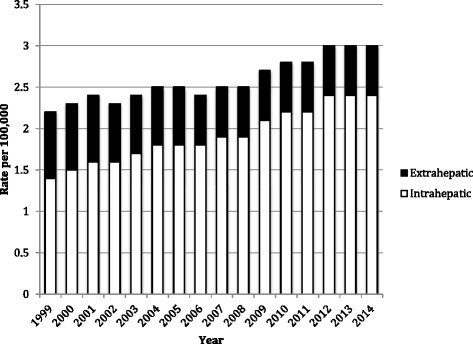
Cholangiocarcinoma mortality for those aged 25+ increased 36 % between 1999 and 2014, from 2.2 per 100,000 (95 % confidence interval [CI] 21-2.3) to 3.0 per 100,000 (95 % CI, 2.9–3.1). Most of the increase resulted from increased intrahepatic cholangiocarcinoma mortality

This study aims to provide CCA mortality rates over time and examine how the rates vary with age, gender, and race. For the time trend analysis (Fig. 1), age-adjusted rates were standardized to US 2000 population using the direct method [16]. The effects of ethnicity/race and gender were derived from Mantel-Haenszel estimates adjusting for age [16]. STATA v.14 (Stata Corp. 2015. version 14. College Station, TX) was used to conduct the analyses.

## Results

This study included 85,248 deaths due to cholangiocarcinoma (CCA). The majority of patients died at age 55 or older (Table 1). The number of CCA deaths increased substantially over time (Fig. 1), from 3889 in 1999 to 7224 in 2014. The age-adjusted CCA mortality rates increased from 2.2 per 100,000 (95 % confidence interval (CI), 2.1–2.3) in 1999 to 3.0 per 100,000 (95 % CI, 2.9–3.1) in 2014. As can be seen in Fig. 1, approximately two-thirds of CCA mortality were from ICC morality, which increased steadily over time; in contrast, ECC mortality stayed relatively stable during the study period.

**Table 1:** Characteristics of patients who died of Cholangiocarcinoma in 1999–2014

Characteristics	*N* = 85,248	%
Age		
25–54	9340	11.0
55–80	62,487	73.3
85+	13,421	15.7
Race		
White	73,533	86.3
African Americans	7144	8.4
Asian or Pacific Islander	4017	4.7
American Indian or Alaska Native	554	0.6
Hispanic origin		
Yes	78,446	92.0
No	6654	7.8
Unknown	148	0.2
Gender		
Male	42,059	49.3
Female	43,189	50.7
Year of diagnosis		
1999–2003	21,301	25.0
2004–2008	24,944	29.3
2009–2014	39,003	45.8

**Fig. 2:**
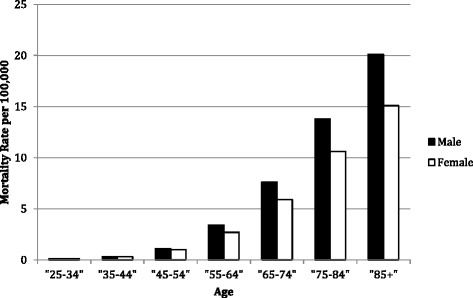
Cholangiocarcinoma mortality increases with age; the risk is higher among males than females. Adjusted risk ratio of females to males is 0.78 (95 % CI 0.77–0.79)

CCA mortality increased with age and has done so even as overall mortality increased. Rates were low for those aged <55 years but increased substantially thereafter, reaching 15.1 (95 % CI 14.7–15.4) per 100,000 among females aged 85+ and 20.0 (95 % CI 19.4–20.6) per 100,000 for males aged 85+ (Fig. 2). In addition to age, gender is an important predictor of CCA mortality: females were at lower risk than males (rate ratio 0.78, 95 % 0.77–0.79).

**Fig. 3:**
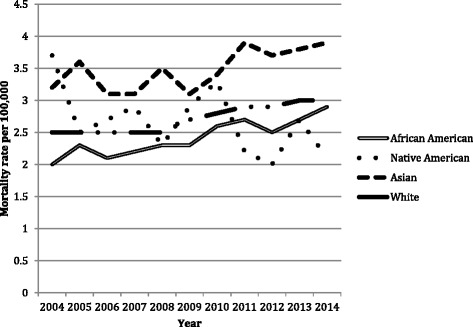
Rate of cholangiocarcinoma mortality decreased among Native Americans but increased in Asians, Whites, and African Americans between 2004 and 2014. The increase in CCA mortality was highest among African Americans (45 %) followed by Asians (22 %) and whites (20 %)

Rate of cholangiocarcinoma mortality decreased among Native Americans but increased in Asians, Whites, and African Americans between 2004 and 2014. The increase in CCA mortality was highest among African Americans (45 %) followed by Asians (22 %), and whites (20 %) (Fig. 3).

**Fig. 4:**
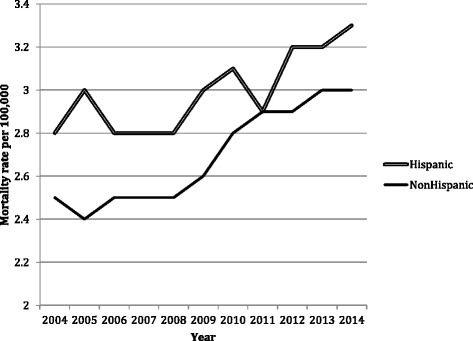
Cholangiocarcinoma mortality increased in both Hispanic and non-Hispanic populations. Between 2004 and 2014, the increase in CCA mortality was 18 % among Hispanics and 38 % among non-Hispanics

Asians were at highest risk of mortality among all races for both men and women. Figure 4 shows that the increase in CCA mortality was 18 % among Hispanics and 38 % among non-Hispanics between 2004 and 2014.

## Discussion

Previous literature is sparse and not conclusive regarding the combined mortality from ICC and ECC. The current study uniquely examines combined ICC and ECC mortality as a group based on the latest national data, and shows a consistent increase in CCA mortality across age and gender since 1999. In our analysis, the increasing trend concentrated in ICC, not ECC. Our study showed that the number of CCA deaths has increased substantially since 1999, reaching over 7000 cases a year in 2013. The majority of patients with CCA develop a recurrence after resection [17], and there is considerable perioperative mortality [18]; therefore, mortality from this disease is a strong indicator of incidence. Our data suggest that the widely quoted rate of 2000–3000 new cases of CCA per year is an under-estimate [19–23], and that there has been a true increase in incidence of CCA. Our results parallel a recent study of data up to 2009 in the 33 Cancer Registries that participate in the North American Association of Central Cancer Registries [12] and are consistent with a trend demonstrated in the incidence of CCA among the SEER population up to 2012 [15], but are derived from more complete national data.

We observed significant variations in CCA mortality across race and gender after adjusting for age distributions. Of particular note, increased risk was associated with male gender overall, Asian ethnicity for both genders, Hispanic women, and advanced age. Notably, while the risk of dying of all cancers combined is highest among African Americans [24], we found that risk of CCA mortality was lower, although increasing, among African Americans compared to other ethnic groups.

The NCHS mortality database does not include data on potential risk factors of CCA such as primary sclerosing cholangitis (PSC), so we cannot quantify the contribution of various risk factors on CCA mortality. However, external data sources show that the higher risk of CCA mortality in males is likely to be related to increased risk in hepatitis C, cirrhosis and PSC. Epidemiological studies have found that risk of CCA increases 27-fold with cirrhosis (adjusted odds ratio, 27.2; *P* <.0001) [25], six-fold with hepatitis C virus infection, (adjusted odds ratio, 6.1; *P* <.0001) [25, 26], and 1560 times with PSC (HR 1560; 95 % CI = 780, 2793; *p* <0.0001) [27]. Furthermore, compared with females, males have higher risk of hepatitis C (176 vs. 105 per 100,000) [28], chronic liver disease (305 vs 206 per 100,000) [28], and PSC (0.45 vs. 0.37 per 100,000) [29]. The higher risk of CCA mortality among males in our study may be related in part to the fact that men are at higher risk for both cirrhosis and PSC [6]. PSC is, however, is a rare diagnosis affecting at most 16.2 per 100,000 people in some studies.

Extensive evidence implicates inflammation and cholestasis as key factors in the pathogenesis of CCA [8, 30, 31]. Inflammatory markers are also universally elevated in metabolic syndrome [32]. In the face of the increasing burden of CCA we have demonstrated and given the increasing incidence of the metabolic syndrome in the United States [33], a recent large study examining preexisting metabolic syndrome as a risk factor for primary liver tumors (not differentiating between ICC and HCC) bears attention [34]. In addition, obesity itself is becoming established as an independent risk factor for CCA [35]. While there are multiple mechanisms by which the metabolic syndrome may be linked to the pathogenesis of gastrointestinal malignancies [36], a putative etiologic link between obesity and CCA is leptin, the hormone regulating homoeostasis which is increased in obese patients, and has been shown to stimulate grown, migration and prevent apoptosis of a CCA cell model [37]. Thus, the underlying etiology of this increase in ICC across gender and race may possibly be related to the prevalence of metabolic syndrome and obesity [34, 35, 37, 38].

In addition to inflammatory risk factors that may cross ethnicities, there are well-established risk factors for CCA specific to ethnicities that merit notice given the significant racial and ethnic variations in CCA mortality in our study. The high risk of CCA mortality among Asians is unsurprising as CCA is more common in Southeast Asia [39, 40], potentially because of the prevalence of infections such hepatitis B and C virus [41], and hepatobiliary fluke infection, prevalent in Asia [42], both inflammatory [43] risk factors for CCA [3, 8, 44–46]. In addition, hepatolithiasis is more commonly noted in Asia than in Western countries and is associated with a 10 % incidence of CCA [8, 47–49]. Another potential contributing factor to the increased incidence among Asians is Type 2 diabetes mellitus (T2DM) [46]. Diabetes has been associated with CCA in a Taiwanese population for both ICC (OR = 2.0) and ECC (OR = 1.8) [46]. The mortality and incidence rate from T2DM increased more in Asians than in their Caucasian or African American counterparts [50], despite on average a substantially lower BMI in the Asian population [51]. African Americans have 2.6 times the mortality due to diabetes compared with those of Asian descent [50], and although the mortality and incidence rates for most other cancers are higher for African Americans [24, 52], and African Americans have the highest increase in CCA mortality, they have substantially lower overall risk of CCA mortality in our study and in the earlier study of ICC [53]. That study also found that although hepatobiliary cancers were highly prevalent in Asian Pacific Islanders, the prevalence of ICC was not significantly different from that of other racial groups [53]. Those results, in combination with the data we present here, suggest that the increased mortality in Asians in the United States may be due to ECCs.

Another notable finding in our study was that the rate of death from CCA increased substantially with age (Fig. 2). This finding is consistent with the trend observed in the SEER databases for HCC, which notes that among persons 75 to 84 years, increases in HCC incidence were seen among all men and white women (*P* ≤0.05) in the United States from 1975 to 2005 [54]. Given that some authors have found decreased mortality from ICC, and have attibuted the decrease to better detection [14], the increase in CCA mortality with age may be in part related to surgical mortality. The incidence of severe and non-surgical postoperative complications is higher in older compared to younger patients undergoing hepatic resection [55] and in patients undergoing surgery for Klatskin Tumors [56]. Because of changes in the US population, this age-dependence may also contribute to increase in total CCA deaths.

In contrast to our findings in CCA, in a review of HCC from the SEER registries and liver cancer mortality data from the National Center for Health Statistics, Altekruse et al. found that HCC incidence rates in SEER registries did not significantly increase during 2007–2010 [57] but the US liver cancer mortality rates did increase [57]. These results suggest that the increased liver cancer mortality in SEER registries may be driven by CCA, not HCC (since ICC was combined with HCC in the SEER registries).

The results of this study must be interpreted in the context of some limitations. First, given that the NCHS mortality database does not include data on comorbidities that are potential risk factors for CCA such as PSC, we cannot quantify the contribution of these risk factors on CCA mortality. Secondly, the mortality data was extracted from death certificates and misclassification might occur in some cases [58, 59]. Misclassification of the cause of death on the death certificate may occur between CCA, pancreatic cancer, gallbladder cancer and hepatocellular cancer [60]. Another potential source of error is that the liver is a common site of adenocarcinoma metastasis, and thus some secondary liver cancers could be mistakenly over-counted as primary liver adenocarcinoma, or ICC [57]. Conversely, CCA can be misdiagnosed as a metastatic adenocarcinoma to the liver rather than a primary liver cancer [15, 61].

Our study has major strengths including the use of recently updated nationally representative NCHS data through 2014 and the provision of data on both ICC and ECC mortality. The inclusion of the entire US data minimizes potential selection bias or referral bias that are commonly encountered in institution-based studies or age limitations of the SEER-Medicare database [25]. Furthermore, the large sample provides a unique opportunity to evaluate how CCA mortality varies with age, gender, and race.

## Conclusions

We found a 36 % increase in CCA mortality in 1999–2014. ICC cases accounted for about three-quarters of all CCA cases, in contrast to earlier reports [3], and showed substantial increase during the study period; ECC mortality has stayed relatively constant during the same period. Older age, being male or Asian is associated with increased risk of CCA mortality. Among different race/ethnic groups, African Americans have the highest increase in CCA mortality.

Understanding and defining the determinants of the ethnic and gender differences informs clinical practice, as they are relevant to developing effective strategies for the prevention, early detection and management of CCA.

## Additional files

Additional file 1: CCA Mortality Rate by Year among Asians; raw data file with query terms. (TXT 6 kb) Additional file 2: CCA Mortality Rate by Year among Hispanics; raw data file with query terms. (TXT 8 kb) Additional file 3: CCA Mortality Rate by Year among Non-Hispanics; raw data file with query terms. (TXT 7 kb) Additional file 4: Excel Spreadsheet raw data used to generate Figure 1. (XLSX 52 kb) Additional file 5: Excel Spreadsheet raw data used to generate Figure 2. (XLS 35 kb) Additional file 6: Excel Spreadsheet raw data used to generate Figure 3. (XLSX 42 kb) Additional file 7: Excel Spreadsheet raw data used to generate Figure 4. (XLSX 12 kb)
